# Marathon related death due to brainstem herniation in rehydration-related hyponatraemia: a case report

**DOI:** 10.1186/1752-1947-1-186

**Published:** 2007-12-28

**Authors:** Axel Petzold, Geoffrey Keir, Ian Appleby

**Affiliations:** 1 The Tavistock Intensive Care Unit, The National Hospital for Neurology and Neurosurgery, Queen Square, London, WC1N 3BG, UK; 2 The Department of Neuroimmunology, The Institute of Neurology, Queen Square, London, WC1N 3BG, UK

## Abstract

**Introduction:**

Identifying marathon runners at risk of neurological deterioration at the end of the race (within a large cohort complaining of exhaustion, dehydration, nausea, headache, dizziness, etc.) is challenging. Here we report a case of rehydration-related hyponatraemia with ensuing brain herniation.

**Case presentation:**

We report the death of runner in his 30's who collapsed in the recovery area following a marathon. Following rehydration he developed a respiratory arrest in the emergency room. He was found to be hyponatraemic (130 mM). A CT brain scan showed severe hydrocephalus and brain stem herniation. Despite emergency insertion of an extraventricular drain, he was tested for brainstem death the following morning. Funduscopy demonstrated an acute-on-chronic papilledema; CSF spectrophotometry did not reveal any trace of oxyhemoglobin or bilirubin, but ferritin levels were considerably raised (530 ng/mL, upper reference value 12 ng/mL), consistent with a previous bleed. Retrospectively it emerged that the patient had suffered from a thunderclap headache some months earlier. Subsequently he developed morning headaches and nausea. This suggests that he may have suffered from a subarachnoid haemorrhage complicated by secondary hydrocephalus. This would explain why in this case the relatively mild rehydration-related hyponatremia may have caused brain swelling sufficient for herniation.

**Conclusion:**

Given the frequency of hyponatraemia in marathon runners (serum Na <135 mM in about 13%), and the non-specific symptoms, we discuss how a simple screening test such as funduscopy may help to identify those who require urgent neuroimaging.

## Introduction

Rehydration-related hyponatraemia and immediate death from brainstem herniation after a marathon is exceedingly rare [1]. In contrast, the risk of acute rehydration-related hyponatraemia (Na<135 mM) in marathon runners is frequent (about 13% [2]). The classical symptoms of acute hyponatraemia are non-specific and comprise lethargy, nausea/vomiting, irritability/restless, disorientation, headaches and muscle weakness/cramps [2,3]. In severe cases drowsiness/confusion, psychosis, seizures, depressed reflexes, neurogenic pulmonary oedema, cerebral infarction and respiratory arrest may develop. Ultimately, brain oedema, herniation and brainstem death occur. To the best of our knowledge there are only two reports of marathon runners in whom brainstem herniation due to hyponatraemic encephalopathy was the suspected cause of death, but neither of these cases was sufficiently well documented in the medical literature to allow for discussion of the clinical presentation and signs needed for further teaching [1,4]. Here we present the first detailed report of a case of rehydration-related hyponatraemia with brain herniation in a marathon runner, and give a didactic discussion of the core clinical features needed to be recognised in the Emergency Room.

## Case presentation

**Figure 1:**
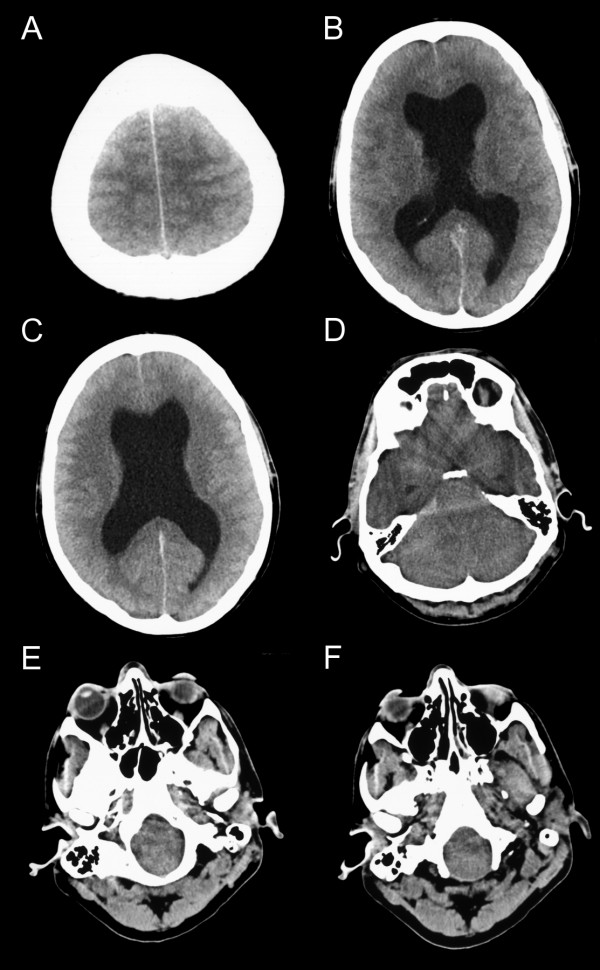
**CT brain scan signs of hydrocephalus, high intracranial pressure and brain stem herniation**. Brain CT (axial slices) in a male patient in his 30's who died of brain stem herniation after completing a marathon. The CT shows (A) loss of the rostral cerebral sulci suggesting increase in ICP, (B) and (C) a large hydrocephalus with widening of both temporal horns. The grey matter can still be differentiated from the white matter, but all sulci are lost. This suggests that the brain oedema is of relative recent onset and massive tissue ischaemia has not yet occurred. (D) Compression of the fourth ventricle with dilatation of the third ventricle and the caudal aspect of both temporal horns. This is observed with considerable brain oedema and obstructive hydrocephalus. (E) Herniation of the medulla and pons into the foramen magnum. (F) The tonsils are located at the level of the dens which is a good indicator for foramen magnum herniation. (All images are from the case presented here).

A male aged in his 30's was admitted to the Emergency Room following collapse in the recovery area following a marathon. He had completed the marathon within around 4 hours on a sunny but cold day. In the Emergency Room he felt faint and dizzy and complained of a headache. His GCS was 15/15, pupils were reactive and his general medical examination was normal. He had a blood pressure of 130/70 mmHg, the ECG showed sinus rhythm of 80 bpm, his chest X-ray did not show any evidence for pulmonary oedema. His blood glucose was 5.4 mM and the Na was 133 mM. He was one of hundreds of athletes presenting at the same time with very similar symptoms, thought to be related to dehydration, and consequently received intravenous rehydration (1 L of 5% Dextrose and 1 L 0.9% NaCl). Four hours after his initial collapse he suddenly vomited and his GCS dropped to 11/15. Shortly after this he suffered a respiratory arrest requiring tracheal intubation. An urgent CT scan showed midbrain herniation into the foramen magnum (Figure 1E and 1F) and severe hydrocephalus (Figure 1A–D), but no fresh blood. An emergency blood screen showed a mild hyponatraemia (Na 130 mM) [2], thought to be due to excessive rehydration. Serum osmolarity was 279 mosmol/kg and urine osmolarity 126 mosmol/kg with normal serum urea (5.0 mM). Serum CK was elevated to 948 IU/L thought to be due to the strenuous exercise. He was then transferred to a nearby neurosurgical centre where an extraventricular drain was inserted for emergency management of high ICP due to hydrocephalus. The ICP was not measured, but the CSF came out under high pressure. On subsequent admission to the intensive care unit (8 hours after his collapse in the recovery area) his pupils were noted to be fixed and dilated and funduscopy demonstrated an acute-on-chronic optic disc oedema [5]. The pupils remained fixed and his GCS was 3/15 off sedation. He underwent formal testing for brainstem death 8 hours after the sedation had been turned off. A postmortem examination was not performed.

A CSF sample was taken during the operative procedure and sent for spectrophotometric assessment of pigments to evaluate whether this could be a CT-negative SAH with secondary hydrocephalus. CSF spectrophotometry did not reveal any trace of oxyhaemoglobin or bilirubin [6], but ferritin levels were considerably raised (530 ng/mL, upper reference value 12 ng/mL) consistent with a previous bleed [7]. Together, these findings suggested that a subarachnoid hemorrhage, complicated by secondary hydrocephalus may have occurred prior to the race. On further questioning of his widow it emerged that the patient had experienced a severe headache three months earlier following an increase in his running schedule. The headache became intolerable and prevented him from sleeping. He felt nauseous, vomited and was unable to move his head because of neck pain. The general practitioner who was called out recorded a high blood pressure and administered paracetamol for pain relief. The patient was unable to return to work for 3 days. The headaches continued in a waxing and waning fashion over the following weeks. He started to develop morning sickness, lost his appetite and stopped having breakfast altogether. Despite these symptoms he continued to increase his running schedule. When he completed his first 22 mile run two months later, he experienced another period of severe headache which was attributed to dehydration. Oral rehydration did not help and he continued to feel run-down to a degree which made it impossible for him to return to work for another 2 days. One month later he ran and finished a marathon whereupon he collapsed and died from brain herniation, a likely consequence of hyponatraemic brain swelling on a background of hydrocephalus secondary to a previous subarachnoid hemorrhage (SAH).

## Discussion

This tragic case illustrates several problems which are frequently seen in the Emergency room: hyponatraemia, headaches and nausea.

### Hyponatraemia

Rehydration-related hyponatraemia occurs in about 13% of athletes [2] making it a potentially difficult logistic problem (with tens of thousands of runners participating in high-profile marathons worldwide). The development of symptoms in acute hyponatraemia depends on the rate of fall of serum Na rather than the absolute degree of hyponatraemia [3]. The osmotic gradient produced between the blood and the brain parenchyma may cause potentially lethal cerebral oedema by increasing the intracranial pressure, leading to tentorial herniation, depression of the respiratory centre and death [3,4,8-10]. In presence of normal blood glucose levels there is no reason to give 5% dextrose (as happened in the present case) because the risk of brain oedema in the presence of hyponatraemia is increased. The level of hyponatraemia in the present case was relatively mild (130 mM) compared to other reported cases of hyponatraemic encephalopathy (Table 1). However, there are important differences in comparison with our case. Firstly, the patient reported by Garigan et al. [9] and by O'Brien et al. [10] was severely fluid-overloaded (9.46 liters of pure water over 90 minutes at one point) and arrived in the Emergency Room with a florid pulmonary oedema [9]. Our patient received only 3 liters of isotonic solutions over 4 hours. Admittedly, the amount of fluid intake during the 4 hour run was not known, but his normal haematocrit on admission of 0.373 and his normal chest X-ray suggests that he was not in fluid overload. The other patient, reported by Ayus et al. [4], died primarily because of mismanagement. She was treated with fluid restriction, a strategy for which there is no supporting data (A Arieff, personal communication).

**Table 1:** Death due to brainstem herniation in rehydration related hyponatremia.

Reference	Gender	Age (years)	Race	Activity	Na (mM)	Presentation
[1]	F	28	Equatorian	Marathon	---	Said she felt dehydrated, rubber-legged and fell to the pavement. She received rehydration. The time to brainstem herniation was not published. She lost consciousness prior to admission and died in hospital the following day.
[9,10]	M	18	Alaska native (Inuit, Yupik)	Military marksmanship training at a temperature of 1190 F (43 C).	121	Dizziness, throbbing headache and nausea. With aggressive rehydration (at one stage, 10 U.S. Quarts/9.5 liter in 90 minutes) he started to vomit. Within four hours from the first symptoms, fixed and dilated pupils were recorded. A chest X-ray showed pulmonary oedema. In intensive care he developed sepsis and disseminated intravascular coagulation and died several days later of cardiac arrest. The postmortem showed diffuse cerebral and brainstem oedema, pituitary infarction [9] and hydrocephalus [10]. (Reference 9 and 10 refer to the same patient. Dr Karen O'Brien, personal communication)
[4]	F	32	---	Marathon^1^	117	Details on symptoms or time course not published^2^. She developed nephrogenic diabetes insipidus and ws treated with fluid restriction. She died of cardiac arrest due to brainstem herniation. The autopsy confirmed brainstem herniation and showed pituitary infarction. (Dr Allen Arieff, personal communication)
Present	M	In his 30's	Caucasian	Marathon	130	Light-headedness and headaches. After rehydration he started to vomit and afterwards suffered a respiratory arrest. The CT brain scan showed midbrain herniation into the foramen magnum and severe hydrocephalus (Figure 1A&B). Formal brainstem death testing was performed 16 hours after he collapsed.

^1^The authors report on 7 patients participating in several marathon runs in Texas, California and Canada between 1993 to 1999. Six patients survived, one died.

^2^From the 7 reported cases, the diagnosis of hyponatremic encephalopathy was suspected in 6 who were treated with intravenous NaCl. All made a full recovery [4]. The patient who died did so primarily because of mismanagement. She was treated with fluid restriction, a strategy for which there is no supporting data (Dr A Arieff, personal communication).

We therefore suspect that our patient may have already suffered from a SAH, producing substantial hydrocephalus prior to the race, thus allowing for only for a small degree of parenchymal brain swelling leading to herniation. It has been suggested that hyponatraemic encephalopathy be named Varon-Ayus syndrome [11].

### Headaches

Any newly developing severe headache is suspicious. A SAH should be suspected if the headaches are of sudden onset and associated with vomiting, meningism (neck stiffness) and a rise in blood pressure. All of this was noted in our case. Characteristically 50% of patients having a SAH experience an instantaneous, thunderclap headache and about 20% will recall similar headaches in the preceding days [12]. Meningism is a good clinical sign if present but it is not sensitive, as it can take hours to develop [13]. Hypertension is a common finding in SAH and understood to be, at least in part, a compensatory phenomenon maintaining cerebral perfusion pressure. If the CT brain imaging does not show any fresh blood, a lumbar puncture for analysis of CSF pigments by spectrophotometry is recommended [6,14]. The results in this case suggest that the recommended analysis of CSF pigments (i.e. bilirubin) may be normal if the bleed occurred more than three weeks ago. CSF bilirubin rises 6–12 hours after a bleed and has been shown to be a very sensitive (100%) marker for up to two weeks following an angiographically proven aneurysmal SAH. Sensitivity decreases to 91% after three weeks and to 71% after four weeks [15]. In these cases the additional measurement of CSF ferritin levels may be of diagnostic value [7,14]. The development of ventricular dilatation following a SAH can be observed in up to 25% of patients. A proportion of these need external ventricular drainage and some will require permanent shunt insertion [13].

### Nausea

**Figure 2:**
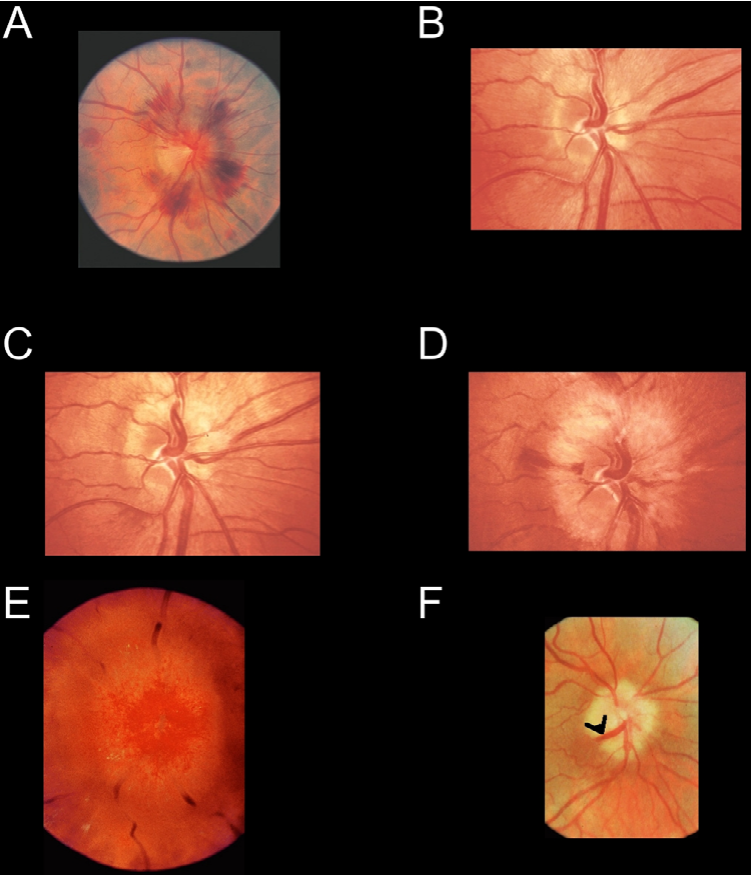
**Funduscopic signs of high intracranial pressure**. (A) The disc shows florid hemorrhages with relatively little swelling, indicating a rapid, dramatic increase in CSF pressure. Progressive changes of optic disc oedema are seen in a patient with an intracranial tumour who declined treatment (B-D). (B) Early nerve fiber dilatation is seen particularly superiorly, inferiorly and nasally. (C) This increases and venous engorgement develops. (D) Temporal nerve fiber dilatation and swelling of the disc increases and hemorrhages appear. (E) In gross chronic disc oedema the normal retinal vasculature is masked and dilated superficial capillaries are observed. (F) In atrophic optic disc oedema nerve fibers are eventually destroyed and the optic disc without viable nerve fibers does not swell. This patient had longstanding benign intracranial hypertension. Retinochoroidal venous collaterals are present (black arrowhead). (All images are reprinted from reference 17, with permission).

Raised intracranial pressure (ICP) should be suspected if nausea (morning sickness) is associated with headaches, loss of appetite and chronic optic disc oedema. The development of optic disc oedema can be classified into four stages: early, developed, chronic and atrophic [5]. Early optic disc oedema can appear 3–4 hours after ictus [16] and a dramatic, acute rise of ICP may even result in peripapillary retinal nerve fibre layer (NFL) hemorrhages with relatively little swelling of the optic disc, as demonstrated in Figure 2A[17].

Probably the most reliable clinical signs of optic disc oedema are swelling of the NFL, peripapillary retinal NFL hemorrhages and blurring of the peripapillary NFL (Figure 2B). The swelling of the NFL also leads to the typical appearance of vessels describing a loop as they emerge from the optic canal (Figure 1C) and obscuration of the retinal vasculature (Figure 2D). In gross optic disc oedema the retinal vasculature may be almost completely masked (Figure 2E). If optic disc oedema persists for a long time, disc swelling disappears as axons degenerate (Figure 2F). Due to the chronically raised pressure in the central retinal vein, retinochoroidal venous collaterals develop (Figure 2F) thereby shunting blood into the choroidal circulation, which has a lower venous pressure [17].

Because of individual differences, retinal hyperemia and blurring of the disc margins are less reliable clinical signs and it is most unlikely that one will have seen (and remember) a particular disc of a patient who presented to the Emergency Room in the past. Typically, acute optic disc oedema arises bilaterally and is associated with full visual fields, normal visual acuity and color vision. The only finding may be an enlarged blind spot [17].

In contrast to acute optic disc oedema, absent spontaneous venous pulsation (SVP) is an unreliable sign for high ICP, because SVP is only observed in 80% of normal subjects [18].

### CSF analysis

In this case the CSF analysis provided important diagnostic clues. Firstly spectrophotometry did not show any trace of haemoglobin, oxyheamoglobin or bilirubin. This virtually excluded a recent bleed [14]. The normal CSF protein of 0.55 g/L (the normal range in our institution is 0.15–0.64 g/L) suggested that there has been no significant breakdown of the blood-CSF barrier. A minor degree of blood-CSF barrier dysfunction cannot be excluded, requiring paired measurement of CSF and serum albumin. Because in this case the ventricular CSF was sampled after insertion of an EVD, procedure-related contamination of the CSF with albumin could have been expected (CSF red cells were 2*10^3^, no CSF white cells were seen). This rendered the CSF:serum albumin ratio as an indicator for integrity of the blood-CSF barrier questionable. Therefore we did not measured the CSF albumin in this case. The high CSF lactate of 12.7 mM (normal serum lacate 1.3 mM) together with the normal CSF total protein and glucose (CSF 2.1 mM, serum 7.9 mM) suggested an increased anaerobic metabolism, possibly due to high ICP and poor CNS perfusion.

The key result in this case was the 44-fold elevated CSF ferritin of 530 ng/mL. It has previously been shown that CSF ferritin, which is too large to pass through the blood-CSF barrier (450–480 kDa), is produced intrathecally [7,19]. CSF ferritin rises primarily in response to a bleed such as a SAH, a stroke with haemorrhagic transformation, or any other form of an intracerebral bleed including superficial siderosis. Elevated ferritin levels have also been observed with CNS necrosis, vasculitis, infections and in miscellaneous CNS infections [14]. Two independent studies showed a significant rise of CSF ferritin levels within 3 days of a SAH [20,21]. Suzuki et al. presented the pooled data for days 3–4 following the bleed with mean CSF ferritin levels of around 250 ng/mL in patients without hydrocephalus and 1000 ng/mL in patients with secondary hydrocephalus [20]. Our own longitudinal data on 24 patients showed median ventricular CSF ferritin levels of 65 ng/mL on day one raising to 1750 ng/mL on day 11 [21]. There is as yet no data in the public domain with regard to the long term (months to years) CSF ferritin levels following a bleed but, in the absence of any complications such as a hydrocephalus, one would expect them to return to normal as the toxic iron is removed.

In summary, a targeted CSF analysis consisting of at least cytology, CSF total protein, glucose, lactate, CSF spectrophotometry and CSF ferritin levels can provide important clues for the diagnostic work up of patients presenting to the Emergency Room with suspected CNS pathology causing non-specific symptoms such as headaches and nausea.

## Conclusion

In conclusion, a simple clinical test such as funduscopy in the Emergency Room may allow for early identification of those athletes who require neuroimaging. With the benefit of hindsight it may have been possible to have suspected a SAH or the presence of intracranial hypertension on the basis of the clinical signs and symptoms in this case.

## Abbreviations

CT = computer tomorgraphy; CSF = Cerebrospinal fluid; GCS = Glasgow Coma Scale; ICP = intracranial pressure; NFL = nerve fiber layer; SAH = subarachnoid haemorrhage; SVP = spontaneous venous pulsation.

## Competing interests

The author(s) declare that they have no competing interests.

## Authors' contributions

AP obtained consent for publication, examined the patient, performed the CSF analysis and reviewed the literature, obtained permission for publishing the personal communications and wrote the manuscript. GK contributed to the data analysis and edited the manuscript. IA examined the patient, contributed to data analysis and co-wrote the manuscript. All authors read and approved the final manuscript.

## Consent

Written, informed consent for publication was obtained from the next of kin. A copy of the written consent is available for review by the Editor-in-Chief of this journal.

## References

[B1] Smith S Marathon runner's death linked to excessive fluid intake New York Times August 13, 2002.

[B2] Almond CSA Shin AY Fortescue EB Mannix RC Wypij D Binstadt BA Duncan CN Olson DP Salerno AE Newburger JW Greenes DS Hyponatremia among Runners in the Boston Marathon New Eng J Med 2005 352 1550 1556 10.1056/NEJMoa043901 15829535

[B3] Stiefel D Petzold A H2O coma Neurocritical Care 2007 6 67 71 10.1385/NCC:6:1:67 17356195

[B4] Ayus JA Varon J Arieff AI Hyponatremia, cerebral oedema, and noncardiogenic pulmonary oedema in marathon runners Ann Intern Med 2000 132 711 714 10787364 10.7326/0003-4819-132-9-200005020-00005

[B5] Hedges TR Papilledema: its recognition and relation to increased intracranial pressure Surv Ophthalmol 1975 19 201 203 1089322

[B6] Petzold A Keir G Sharpe LT Spectralphotometry for xanthochromia New Eng J Med 2004 351 1695 1696 10.1056/NEJM200410143511627 15483297

[B7] Keir G Tasdemir N Thompson EJ Cerebrospinal fluid ferritin in brain necrosis: evidence for local synthesis Clin Chim Acta 1993 216 153 156 10.1016/0009-8981(93)90148-W 8222266

[B8] Arieff AI Hyponatremia, convulsions, respiratory arrest, and permanent brain damage after elective surgery in healthy women N Engl J Med 1986 314 1529 1535 3713746 10.1056/NEJM198606123142401

[B9] Garigan TP Ristedy DE Death from hyponatremia as a result of acute water intoxication in an Army basic trainee Mil Med 1999 164 238 238 10091501

[B10] O'Brien KK Montain SJ Corr WP Sawka MN Knapik JJ Craig SC Hyponatremia associated with overhydration in U.S. Army trainees Mil Med 2001 166 5 405 410 11370203

[B11] Acosta P Varon J Life-threatening hyponatremia in marathon runners: The Varon-Ayus syndrome revisited Crit Care & Shock 2005 8 23 27

[B12] Linn FH Rinkel GJ Algra A van Gijn J Headache characteristics in subarachnoid haemorrhage and benign thunderclap headache J Neurol Neurosurg Psychiatry 1998 65 791 793 9810961 10.1136/jnnp.65.5.791 PMC2170334

[B13] van Gjin J Kerr RS Rinkel GJE Subarachnoid haemorrhage The Lancet 2007 369 306 318 10.1016/S0140-6736(07)60153-6 17258671

[B14] Petzold A Sharpe LT Keir G Spectrophotometry for cerebrospinal fluid pigment analysis: a review Neurocritical Care 2006 4 153 162 10.1385/NCC:4:2:153 16627907

[B15] Vermeulen M Hasan D Blijenberg BG Hijdra A van Gijn J Xanthochromia after subarachnoid haemorrhage needs no revisitation J Neurol Neurosurg Psychiatry 1989 52 826 808 2769274 10.1136/jnnp.52.7.826 PMC1031927

[B16] Pagani LF The rapid appearance of papilledema J Neurosurg 1969 30 247 249 5780896 10.3171/jns.1969.30.3part1.0247

[B17] Spalton DJ Hitchings RA Hunter PA Atlas of Clinical Ophthalmology 2005 3 Elsevier MOSBY

[B18] Levin BE The clinical significance of spontaneous pulsations of the retinal vein Arch Neurol 1978 35 37 40 619871 10.1001/archneur.1978.00500250041009

[B19] Hallgren R Terent A Wide L Bergström K Birgegård G Cerebrospinal fluid ferritin in patients with cerebral infarction or bleeding Acta Neurol Scand 1980 61 384 392 7415809 10.1111/j.1600-0404.1980.tb01508.x

[B20] Suzuki H Muramatsu M Tanaka K Fujiwara H Kojima T Taki W Cerebrospinal fluid ferritin in chronic hydrocephalus after aneurysmal subarachnoid hemorrhage J Neurol 2006 253 1170 1176 10.1007/s00415-006-0184-1 16649098

[B21] Petzold A Worthington VC Kerr M Kitchen N Smith M Keir G Improving the diagnosis of subarachnoid haemorrhage: the value of cerebrospinal fluid ferritin levels Neurocritical Care 2007 6 244

